# Supplementary material for: A gas-plastic elastomer that quickly self-heals damage with the aid of CO2 gas
Source: Nat Commun. 2019 Apr 23;10:1828. doi: 10.1038/s41467-019-09826-2 (PMC6478687; doi:10.1038/s41467-019-09826-2)
Supplement: Supplementary file 1 — Supplementary Information [file 41467_2019_9826_MOESM1_ESM.pdf]

Supplementary Information for

A gas-plastic elastomer that quickly self-heals damage with the aid of CO_2 gas

Miwa *et al.*

Supplementary Methods

Materials. Diethoxydimethylsilane (DEDMS, >98%), 3-aminopropyldiethoxymethylsilane (3-APDEMS, >97%), and 4-carboxy-2,2,6,6-tetramethylpiperidine 1-oxyl (4-carboxy-TEMPO, >97%) were purchased from Tokyo Chemical Industry Co., Ltd. Molecular sieves (3A 1/16), succinic anhydride (extra-pure reagent), CaH_2 (extra-pure reagent), and NaOH (97%) were purchased from Nacalai Tesque, Inc. Acetone, tetrahydrofuran (THF), chloroform, toluene, and ethanol, all extra-pure regent grade, were obtained from Kanto Chemical Co., Inc. A chemically crosslinked silicone elastomer sheet was purchased from AS ONE Co.

Polymerization of poly(dimethyl siloxane) (PDMS)-NH_2. A mixture of DEDMS (128.9 g, 869 mmol), 3-APDEMS (3.3 g, 17 mmol), ethanol (102 mL, 1.75 mol), and water (104 mL, 5.77 mol) was stirred in a Teflon beaker at room temperature for 24 h using a magnetic stirring bar. The reaction mixture was further stirred at 80 °C for 15.5 h to remove the solvents. The resulting oily sample was heated at 82 °C for 20.5 h and then at 148 °C for 72 h, both in a vacuum. The M_n and M_w/M_n, determined using gel permeation chromatography (GPC) using polystyrene standards (TOSOH Corporation), were 7,300 and 1.56, respectively. The actual amino groups' concentration calculated from ^{1}H-NMR spectra was approximately 2.7 mol%. ^{1}H-NMR (400 MHz, $CDCl_3$) (Supplementary Figure 2A): δ (ppm) = 0.07 (m, 6H), δ = 0.50 (t t, 2H), δ = 1.47 (quint t, 2H), δ = 2.65 (t, 2H).

Carboxylation of PDMS-NH_2. Chloroform was dried for 24 h using molecular sieves (3A) and CaH_2. The dried succinic anhydride (22.7 g) was dissolved in the dried chloroform (400 mL) at 50 °C. Some succinic anhydride was undissolved and precipitated. This saturated solution was poured into PDMS-NH_2 (41.4 g) and stirred at 50 °C for 1 h. The precipitated succinic anhydride was filtered, and the chloroform was removed from the reaction mixture using an evaporator. The reaction mixture was then dissolved in THF and poured into water, and the formed precipitation was isolated. The resultant carboxylic PDMS (PDMS-COOH) was dried under vacuum at 47 °C for more than 39 h. The amide group (ν = 1650 cm^{-1} (C=O)) formation was confirmed using Fourier-transform infrared (FT-IR) spectroscopy (Supplementary Figure 3). The actual COOH group concentration was determined by neutralizing titration with a NaOH/methanol solution using phenolphthalein as the indicator. The COOH group

concentration was 3.8 mol%, which is higher than that of the NH_2 group because succinic anhydride was added to the hydroxy chain ends. ^{1}H-NMR (400 MHz, $CDCl_3$/[D_6]DMSO (90/10 v/v)) (Supplementary Figure 2B): δ (ppm) = 0.07 (m, 6H), δ = 0.50 (t t, 2H), δ = 1.52 (quint t, 2H), δ = 2.46 (t, 2H), δ = 2.58 (m, 10H), δ = 3.18 (q, 2H), δ = 6.53 (s, 1H).

Neutralization of PDMS-COOH and preparation of cast films. An appropriate amount of a NaOH/methanol solution (568 mmol L^{-1}) was slowly added to a PDMS-COOH/THF solution (7 wt%) under vigorous stirring. The solution viscosity increased because the carboxyl groups were neutralized. The mixture was then poured into a Teflon petri dish and dried at 35 °C to make a cast film. The cast film was further dried at 35 °C for more than 1 d in a vacuum. The cast film thickness was approximately 0.4 mm. The FT-IR spectra of PDMS-NH_2, PDMS-COOH, and PDMS-*x*Na are shown in Supplementary Figure 3. Upon neutralization, the intensity of the COOH carbonyl stretching band at 1716 cm^{-1} decreases, and a band indicating sodium carboxylate's symmetric stretching vibration is generated at 1586 cm^{-1}.

Spin probing. The spin probe reagent, 4-carboxy-TEMPO, was dissolved in THF to attain a concentration of 2.0×10^{-4} g L^{-1}. PDMS-80Na (86 mg) was dissolved in THF (2 mL), and 0.5 mL of the spin probe reagent solution was added. The mixture was dried at 35 °C for 2 d and then at room temperature for 2 d, both under vacuum. The sample films were cut in small pieces and encapsulated in 5 mm o.d. quartz tubes for electron spin resonance (ESR) analyses. The tubes were sealed with dry air, CO_2, or N_2 gases followed by evacuation. The concentration of the 4-carboxy TEMPO in the sample was low enough to avoid broadening the ESR spectrum due to spin–spin interactions.

^{1}H-Nuclear magnetic resonance (NMR). ^{1}H-NMR spectra were obtained on JEOL JNM-CX400P (400 MHz) spectrometer. The samples were dissolved in $CDCl_3$ or $CDCl_3$/[D_6]DMSO (90/10 v/v) containing tetramethylsilane as an internal standard.

Gel permeation chromatography (GPC). GPC was performed to determine the weight and number average molecular weights of the PDMS-NH_2 using an HLC-8020 apparatus manufactured by Tosoh Co., Ltd., equipped with two polystyrene gel columns (Tosoh TSKgel GMH, G4000HXL and G2000HXL) connected to a RI-4030 RI

detector (JASCO). THF was used as the eluent at 40 °C. The column set was calibrated using standard polystyrene (Tosoh) samples with small polydispersity indices.

Differential scanning calorimetry (DSC). DSC measurements were conducted using a DSC7020 differential scanning calorimeter, manufactured by SII, and calibrated with indium, zinc, lead, and tin standards. A quench cooler accessory was used to cool the samples. During the measurements, the DSC cell was purged with dry N_2 gas at a flow rate of 50 mL min^{-1}. The samples were heated from −150 to 100 °C at a rate of 10 °C min^{-1}. The midpoint T_g, i.e., the temperature corresponding to half the endothermic shift, was subject to an experimental error of ± 1 °C.

Fourier-transform infrared (FT-IR) spectroscopy. FT-IR spectra were obtained with a Perkin-Elmer Spectrum400 spectrometer equipped with a DTGS detector. A thin film sample was prepared on a KBr plate from a chloroform solution. The film was dried at room temperature for more than 12 h under vacuum before measurement. The sample thickness was controlled to ensure that the bands' absorbances were the Lambert–Beer law range. Measurements were performed in transmittance mode at an optical resolution of 4 cm^{-1} using 32 scans.

Tensile test. The sample films' tensile stress–strain curves were collected using the AND Force Tester MCT-2150 at 27±1°C under various gases (dry air, N_2, and CO_2). Dumbbell-shaped tensile bars, with dimensions of 25 × 2.0 × 0.4 mm, were cut from the cast films. The initial gauge length was typically set to 11 mm. Tensile bars were separately stretched at speeds of 10, 100, and 300 mm min^{-1}. Each measurement was performed at least three times. The tensile stress (σ) was calculated as $\sigma = F/S_0$, where F is the loading force, and S_0 is the initial cross-sectional area of the sample film. The strain (ε) under elongation was defined as the marker distance (l) relative to the initial marker distance (l_0) of the specimen, i.e., $\varepsilon = (l – l_0)/l_0 \times 100\%$. The increasing marker distance was monitored by a video camera. Before tensile testing in each gas, the PDMS-80Na film was exposed to an atmosphere of that gas for 2 h. The moisture-absorbed PDMS-80Na was measured in air. Prior to the measurement, a PDMS-80Na film was stored for 5 days in a glass desiccator maintained at 75 % humidity and 28 °C. The weight of the film increased by 2% because of the moisture absorption.

Dynamic mechanical measurement. Dynamic mechanical measurements were performed in tensile mode on a TA Instruments DMA Q800. Isothermal measurements

were performed in each gas atmosphere at 1 Hz and 30°C. The rectangular specimen dimensions were 10 × 4.5 × 0.4 mm, and a strain of 0.5% was applied, which was within the linear viscoelasticity regime. Each gas (dry air, N_2, and CO_2) was flown into the sample chamber at a rate of 1.6 L min^{-1} during the measurements. Creep measurements were performed with a stress of 4 kPa.

Rheological measurement. To elucidate PDMS-*x*Na's linear viscoelastic properties, the rheological properties were investigated in oscillatory shear on a parallel-plate rheometer (AR-G2, TA instruments) with 8 mm diameter plates. The sample thickness was approximately 0.4 mm, and a strain of 0.5% was applied, which was within the linear viscoelasticity regime. A temperature sweep test was conducted at 1 Hz in –150–150°C range at a heating rate of 3 °C min^{-1}. The sample temperature was controlled with flowing N_2 gas generated from liquid N_2. Frequency sweep tests were performed in each gas atmosphere (dry air, N_2, and CO_2) within a dynamic range from 0.01 to 50 Hz. Before the frequency sweep measurements, the PDMS-80Na film was exposed to an atmosphere of the relevant gas for 2 h. The moisture-absorbed PDMS-80Na was measured in air. Prior to the measurement, a PDMS-80Na film was stored for 5 days in a glass desiccator maintained at 75 % humidity and 28 °C. The weight of the film increased by 2% because of the moisture absorption.

Small-angle X-ray scattering (SAXS). Synchrotron SAXS measurements were performed using the BL-6A beamline at the Photon Factory of the High Energy Accelerator Research Organization (KEK) in Tsukuba, Japan. The X-ray wavelength (λ) was 0.15 nm. A PILATUS-1M was used as the detector and placed 1.0 m away from the sample position. Stearic acid and silver behenate were used as SAXS detector calibration standards. The experimental data were corrected for background scattering and sample absorption. The intensities were radially integrated, averaged, and redistributed to convert the pixel number to the corresponding scattering vector q ($q = (4\pi/\lambda)\sin\theta$) and produce a circularly averaged pattern. Experimental scattering patterns were fitted using the Yarusso–Cooper model[1]. In this model, spherical ionic aggregates, with a radius of R_1, disperse randomly, but are limited by the radius of closest approach, $2R_{CA}$. The scattering intensity for this model is expressed by

$$I(q) = \frac{K}{V_p} V_1^2 \Phi(qR_1)^2 \frac{1}{1+(\frac{8V_{CA}}{V_p})\Phi(2qR_{CA})} \qquad (1)$$

$$V_{CA} = \frac{4}{3}\pi R_{CA}^3 \quad (2)$$

$$V_1 = \frac{4}{3}\pi R_1^3 \quad (3)$$

$$\Phi(x) = 3\frac{\sin x - x\cos x}{x^3} \quad (4)$$

where V_p and K are the average sample volume occupied by one particle of the ionic aggregate and an adjustable parameter for the intensity scale. The upturn at the small angle region in the PDMS-90Na is fitted using a Lorentz function.

Electron spin resonance (ESR). A JEOL X-band (~9 GHz) FA100 spectrometer with 100 kHz field modulation was used for ESR measurements. The experimental parameters include a modulation amplitude of 0.25 mT, a magnetic field width of 20 mT, a sweep time of 30 s, and a time constant of 0.01 s. Two scans were performed. The magnetic field and g tensor were calibrated with a Mn^{2+} standard. The saturated and unsaturated microwave powers used for measurements were 16 and 0.02 mW, respectively. The measurements were performed from −150 to −70 °C at 2.5 °C intervals.

Density functional theory (DFT) calculation. The molecular structures were optimized using the M06 DFT/6-31G** method. We also performed a normal mode analysis, which confirmed none of the optimized structures have imaginary frequencies. The interaction energies were calculated based on the zero-point vibrational energy-corrected DFT energies. All calculations were performed using the GAUSSIAN16 Revision B.01 program package.

Self-healing tests. The self-healing of scratches on the PDMS-70Na film surface was monitored by an optical microscope (Olympus BX53P). The film was scratched using a razor, and the depth of the scratch was controlled to approximately 0.15 mm using a spacer. Scratched PDMS-70Na films were separately mounted on slide glass and stored at room temperature (26°C) in either dry air or a dry CO_2 atmosphere. The strength recovery of the self-healed films was measured via tensile testing at 27±1°C. A PDMS-80Na film was scored, leaving a thickness of 12.5 μm, using a razor and a spacer to avoid completely cutting the film into two separate pieces. The cut faces were then placed in contact. The PDMS-80Na films were stored at room temperature (26°C) in either dry air or CO_2 for different periods. Before the tensile test, the PDMS-80Na film

that self-healed in a CO_2 atmosphere was evacuated for 10 minutes to remove the CO_2. The healed PDMS-80Na films were then stretched at 100 mm min^{-1} in air. The self-healing efficiency was calculated as the ratio between the tension energies required to break the original and self-healed materials. The tension energies were measured as the area below the stress–strain curve.

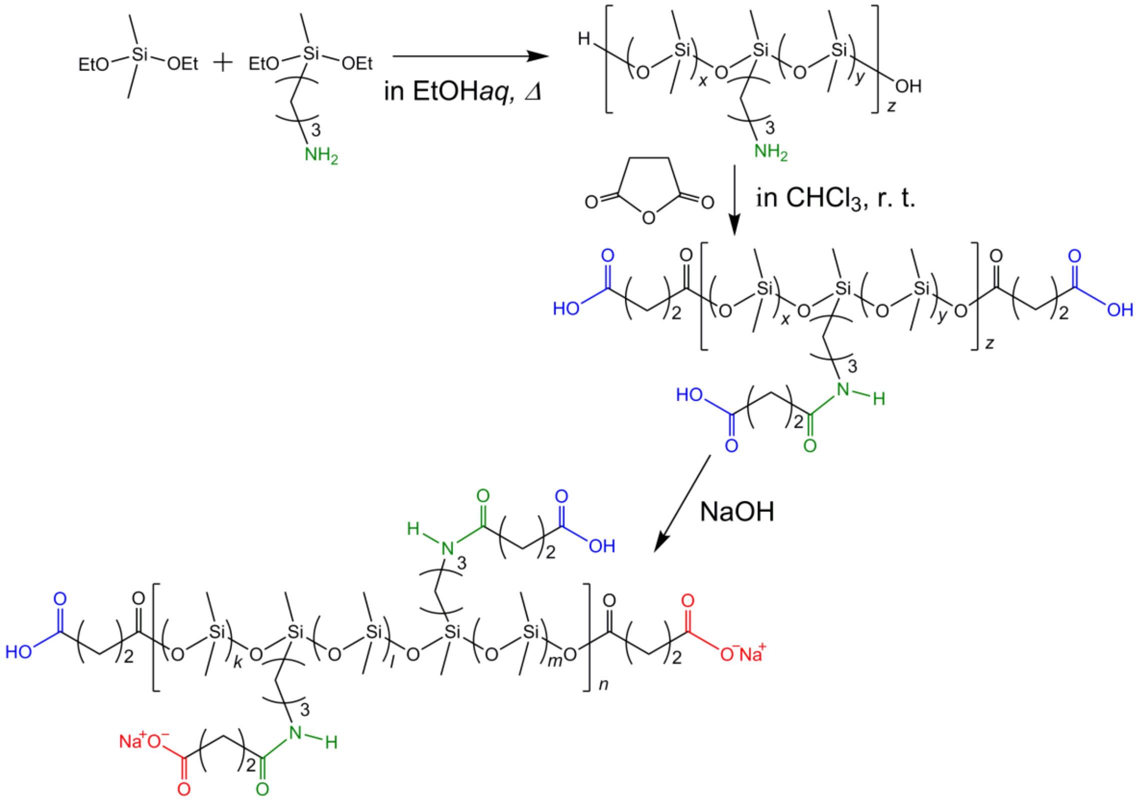

Supplementary Figure 1. Synthesis route for PDMS-*x*Na.

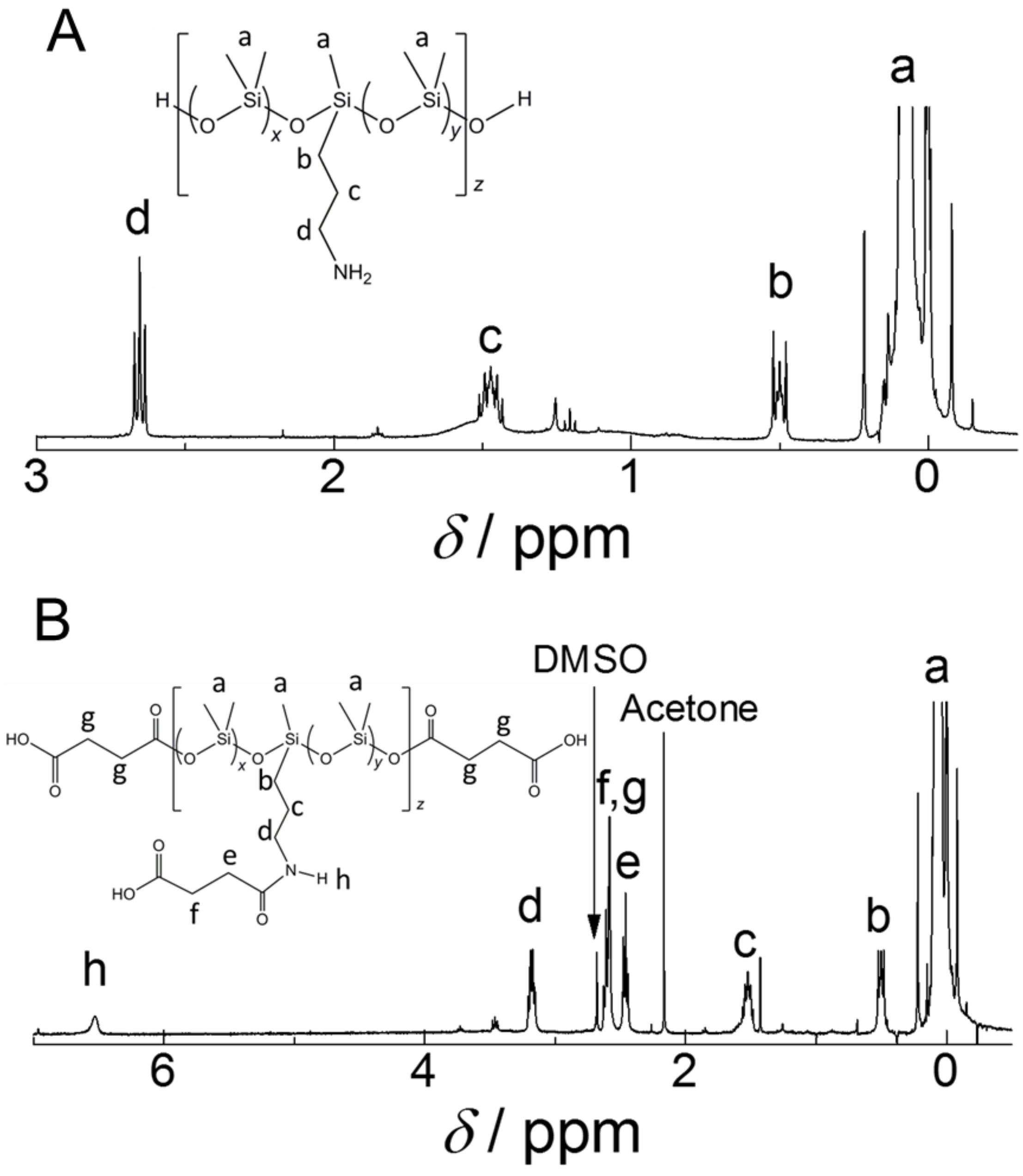

Supplementary Figure 2. ^{1}H-NMR spectra of (A) PDMS-NH_2 and (B) PDMS-COOH.

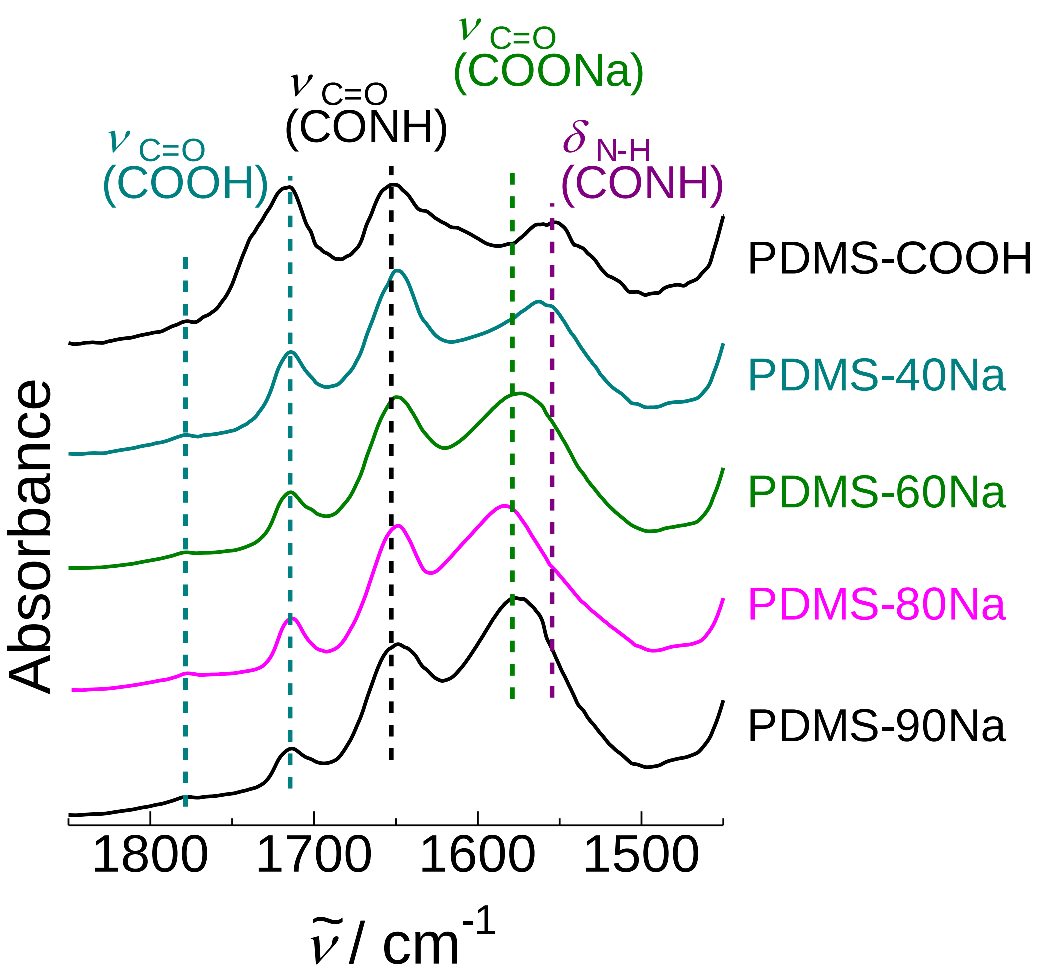

Supplementary Figure 3. FT-IR spectra of PDMS-COOH and PDMS-*x*Na.

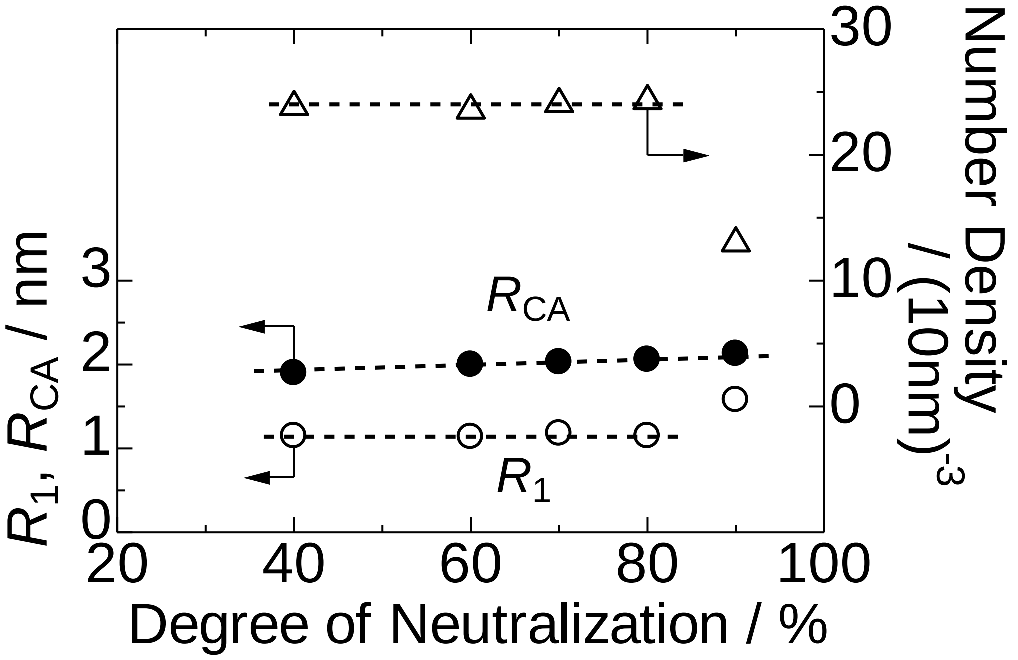

Supplementary Figure 4. SAXS determinations of the sizes (left) and number densities (right) of ionic aggregates in PDMS-*x*Na.

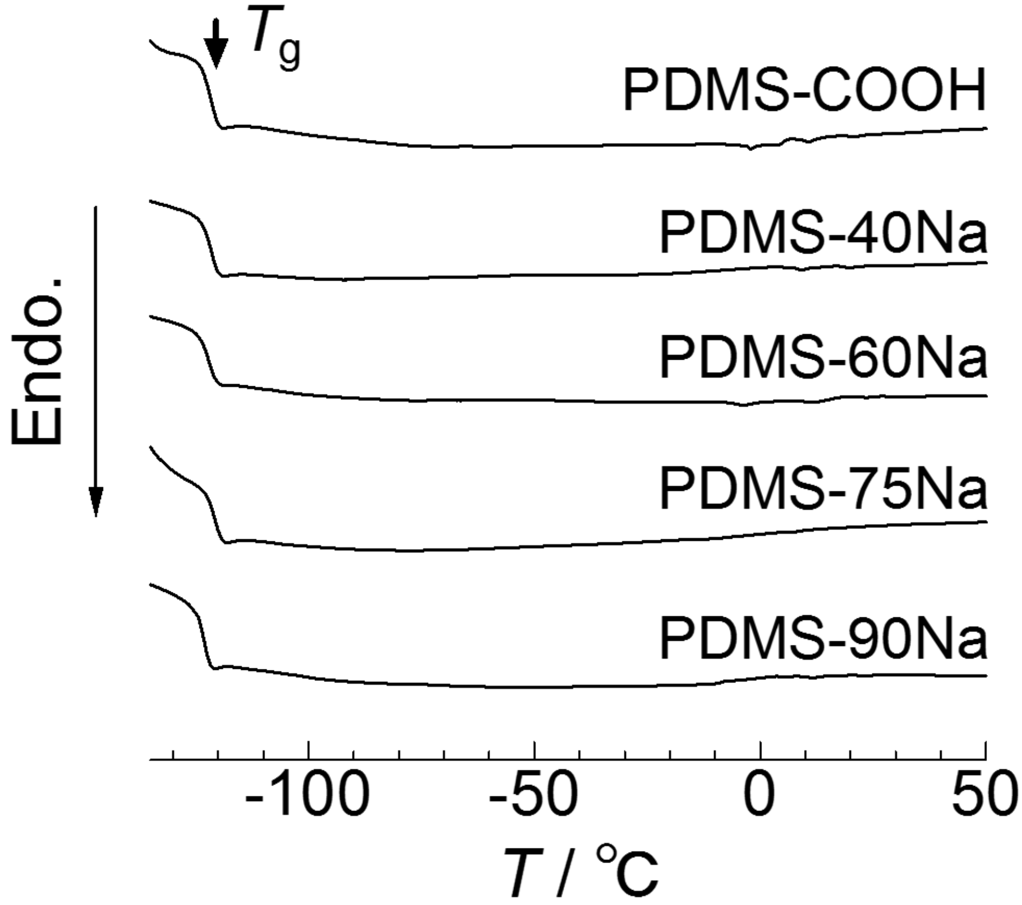

Supplementary Figure 5. DSC traces of PDMS-COOH and PDMS-*x*Na.

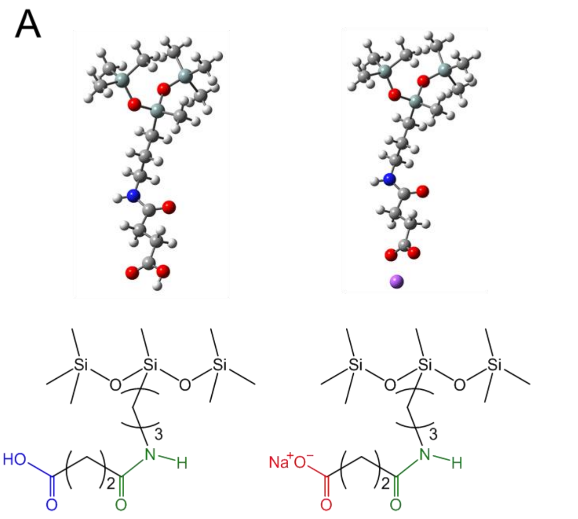

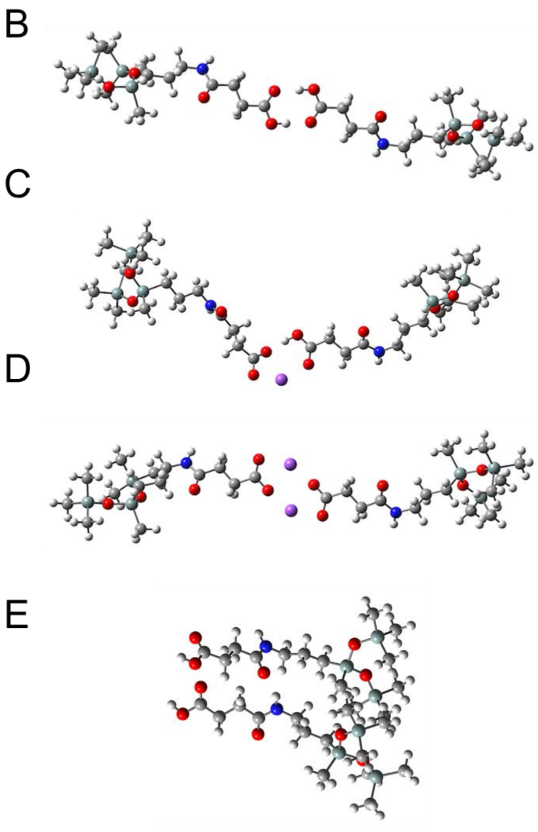

		Interaction energy / $kJ\ mol^{-1}$
B	COOH···COOH	-74.5
C	COOH···COONa	-116
D	COONa···COONa	-190
E	N-H···O=C	-78.7

Supplementary Figure 6. DFT calculation results. (A) Model units used for calculation. (B-E) Stable structures for model unit dimers.

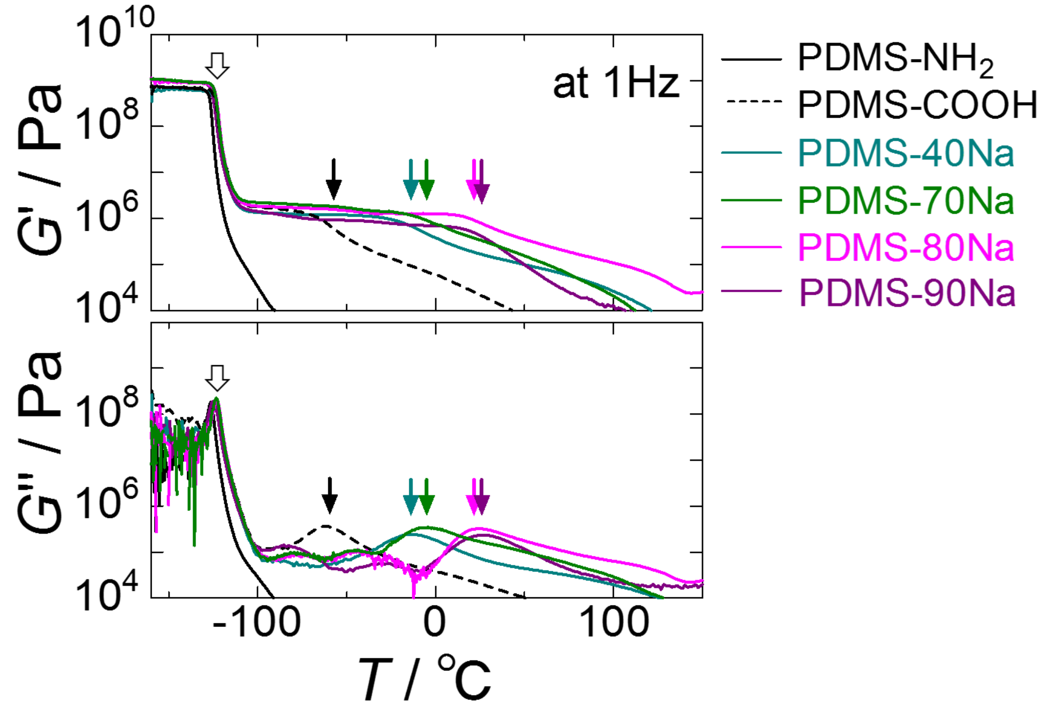

Supplementary Figure 7. Rheological results of PDMS-NH$_2$, PDMS-COOH, and PDMS-*x*Na. The temperature sweep of the storage modulus (G') and loss modulus (G'') measured at 1 Hz. Relaxations assigned to the glass transition and network rearrangement are indicated by open and solid arrows, respectively.

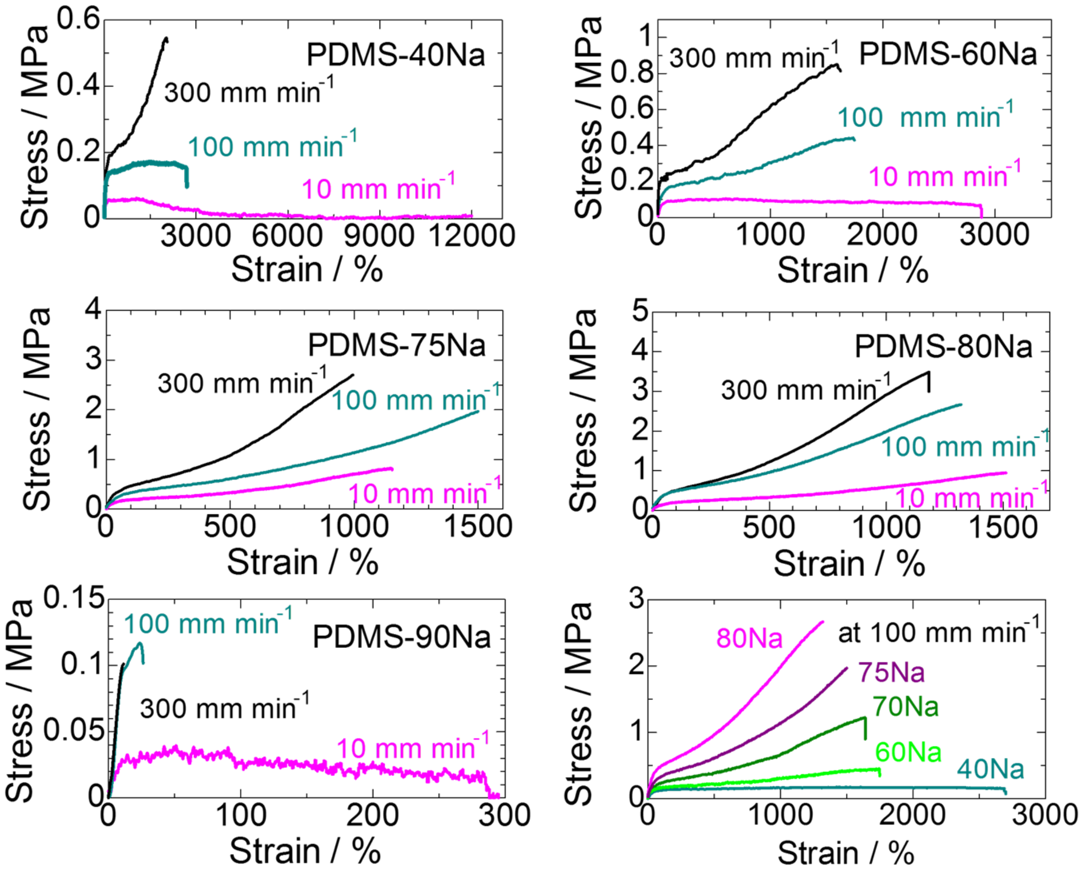

Supplementary Figure 8. Mechanical properties of PDMS-*x*Na. Tensile stress–strain curves of PDMS-*x*Na stretched at different speeds. Bottom right, comparison of PDMS-*x*Na stretched at 100 mm min^{-1}.

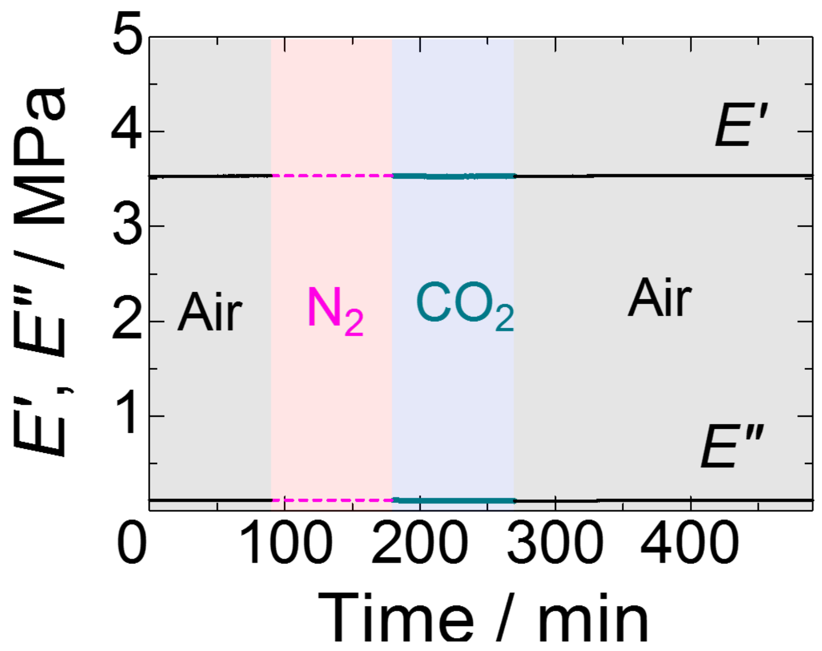

Supplementary Figure 9. Effect of CO_2 on mechanical properties. The effect of each gas on storage modulus (*E*′) and loss modulus (*E*′′) for commercially available, chemically crosslinked PDMS was measured at 1 Hz at 30°C.

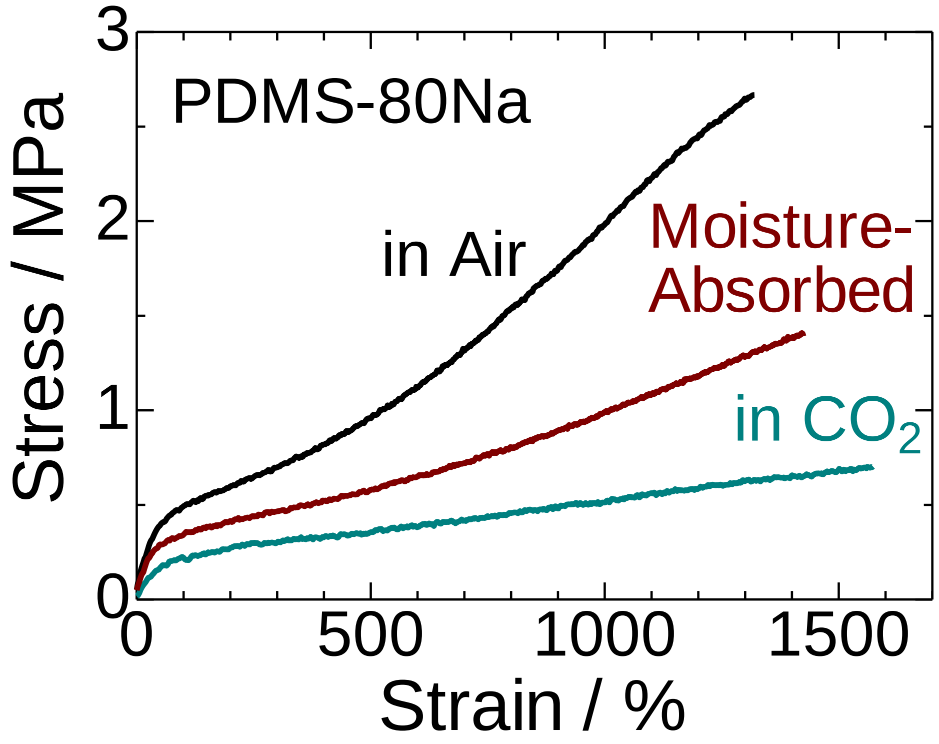

Supplementary Figure 10. Effect of moisture absorption on tensile property. Tensile tests for PDMS-80Na measured at 27 ± 1 °C in dry air and in dry CO_2. The result for moisture-absorbed PDMS-80Na measured in air is indicated with brown curve. The stretching speed is 100 mm min^{-1}.

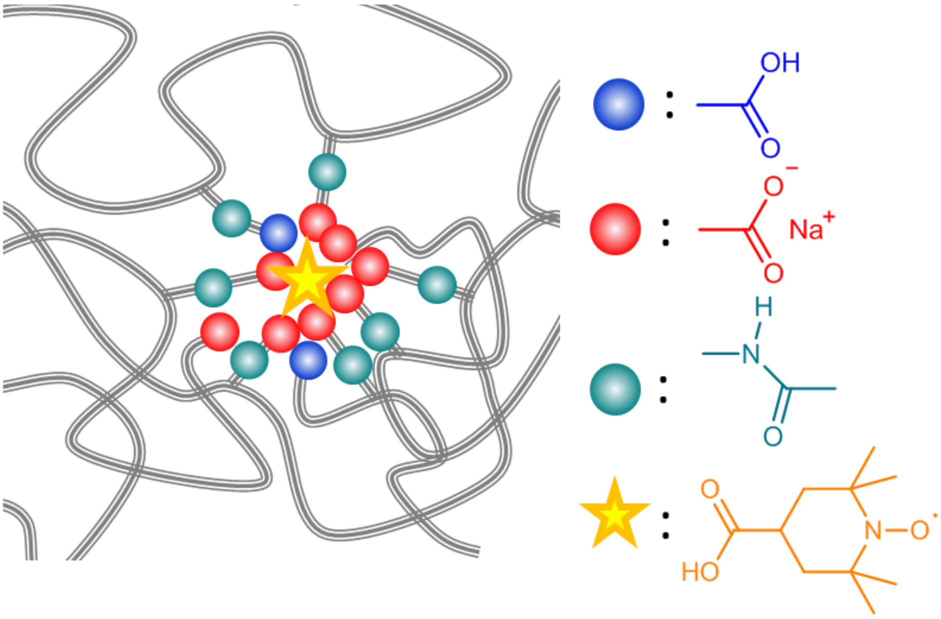

Supplementary Figure 11. Schematic illustration of selective localization of 4-carboxy-TEMPO within ionic aggregate.

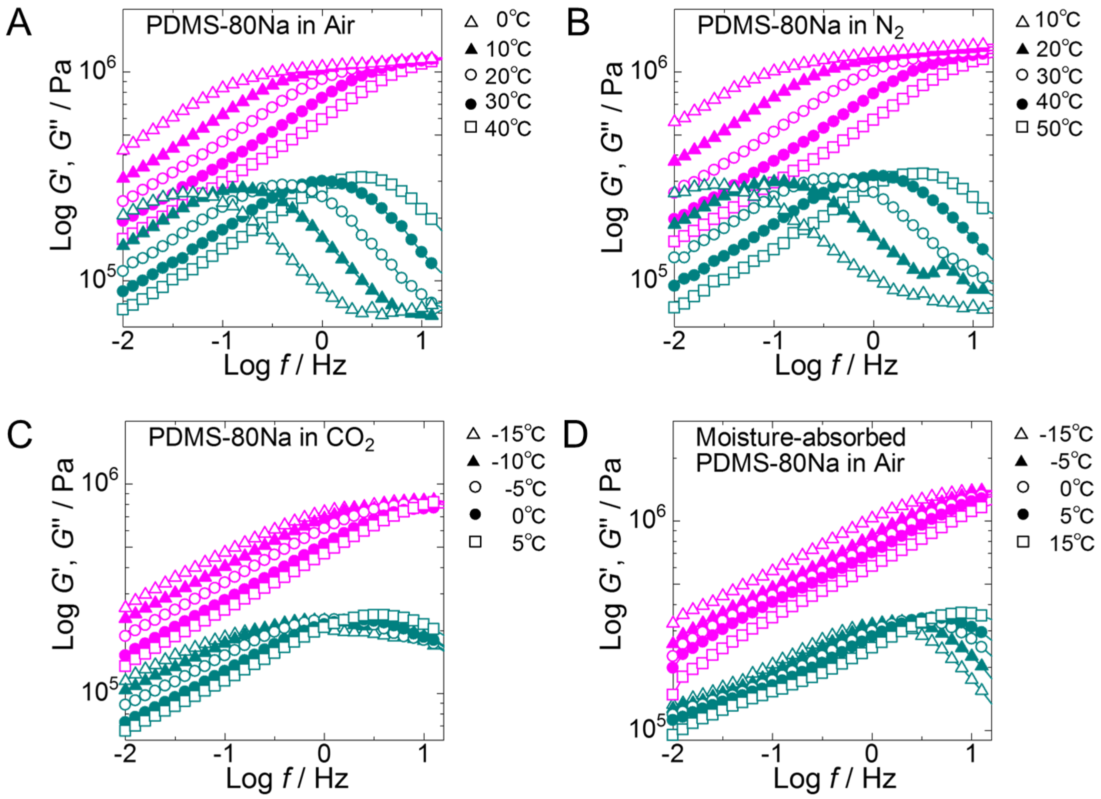

Supplementary Figure 12. Effects of each gas and moisture-absorption on network rearrangement. Frequency sweeps of storage modulus (*G'*, magenta) and loss modulus (*G''*, blue green) of dried PDMS-80Na measured in (A) air, (B) N_2, and (C) CO_2. (D) Result for moisture-absorbed PDMS-80Na measured in air. The relaxation is assigned to the rearrangement of ionic crosslinks.

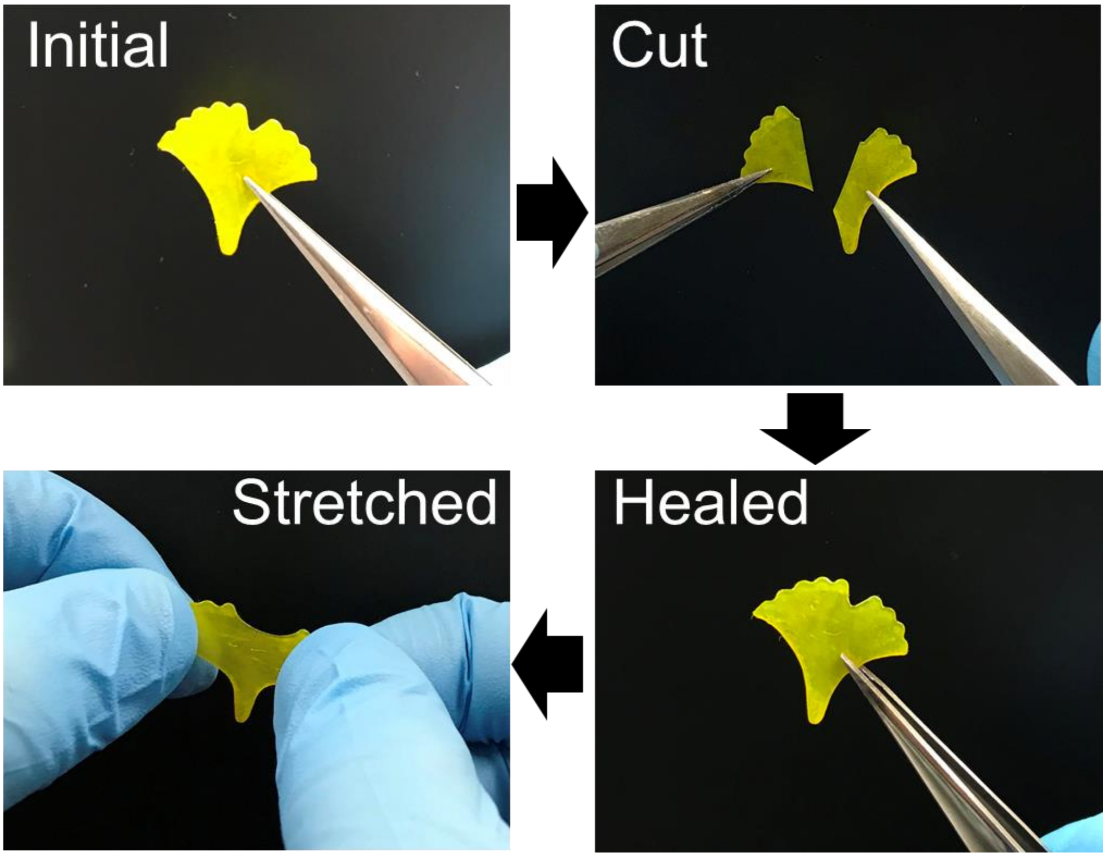

Supplementary Figure 13. Self-healing of PDMS-60Na. Photograph of self-healing behavior of ginkgo-shaped PDMS-60Na film at 28 °C. The film was painted yellow.

Supplementary Reference

[1] Yarusso, D. J., & Cooper, S. L. Analysis of SAXS data from ionomer systems. *Polymer* **26**, 371–378 (1985).
